# Effect of dietary turmeric and cinnamon powders on meat quality and lipid peroxidation of broiler chicken under heat stress condition

**Published:** 2017-06-15

**Authors:** Payam Baghban Kanani, Mohsen Daneshyar, Javad Aliakbarlu, Fatemeh Hamian

**Affiliations:** 1 *Department of Animal Sciences, Faculty of Agriculture, Urmia University, Urmia, Iran; *; 2 *Department of Food Hygiene and Quality Control, Faculty of Veterinary Medicine, Urmia University, Urmia, Iran.*

**Keywords:** Broiler, Cinnamon, Lightness, Radical scavenging activity, Turmeric

## Abstract

Two hundred and fifty male 1-day-old broiler chicks (Ross 308) were used to investigate the effects of dietary supplementation of turmeric and cinnamon powders on meat quality and lipid peroxidation of broilers under heat stress condition. The five treatment groups were control (recommended temperature for Ross 308), heat stressed (32 ± 1 ˚C from 9:00 AM to 5:00 PM during finisher period) and heat stressed birds fed with 0.50% turmeric, 0.50% cinnamon and a blend of cinnamon and turmeric (0.25% turmeric + 0.25% cinnamon). The results showed that there were no significant differences between the treatments for ether extract, ash and crude protein contents of thigh meat at 42 day of age (*p* > 0.05). Heat stress decreased the pH value and dry matter (DM) content of thigh meat, whereas the consumption of all experimental diets (turmeric, cinnamon and both of them) compensated the decreased pH and DM values due to heat stress to some extent but could not restore them to the level of control treatment (*p* < 0.01). Furthermore, the thigh meat lightness was increased under heat stress (*p *< 0.05). The thiobarbituric acid reactive substances and free radicals scavenging activity were increased in thigh meat of broilers reared under heat stress (*p* < 0.05), while these parameters were reduced by the combination of both plants (*p* < 0.01). It was concluded that heat stress reduces antioxidant properties and quality of thigh meat and dietary supplementation of turmeric and cinnamon powders together can remove the detrimental effects of heat stress on meat quality.

## Introduction

The combination of high environmental temperature and humidity affect the performance of poultry in the tropical regions of the world. Higher humidity and environmental temperature above the thermal comfort zone increase the susceptibility of the birds to heat stress and negatively affect the performance and immune system efficiency.^1^ Actions of free radicals and reactive oxygen species (ROS) during stress result in cell wall damage and cell function loss.^1^ The high levels of free radicals production in the body can induce lipid peroxidation and affect the meat quality indices.^2^ Heat stress leads to the induction of oxidative stress and adversely affects the vital functions of the cell such as transcription, RNA processing, translation, metabolism and membrane structure.^3^ Moreover, heat stress possibly decreases the metabolic antioxidant capacity of the body.^4^ Hence, the concentrations of dietary pro-oxidants and antioxidants can affect lipid peroxidation of poultry products.^5^ Therefore, dietary supplementation of antioxidants may help in the oxidation-reduction of poultry products during heat stress conditions.^6^^,7^ The oxidation of lipids is a main cause of meat quality deterioration and possibly functional, sensory and nutritive values reductions of meat products.^8^ This oxidation and production of undesirable odors and flavors consequently decrease the meat acceptability for the consumers.^9^ Furthermore, heat stress results in a faster pH decline and cause a pale color of breast meat in birds. It has been reported that increased lightness (L), lower redness (a) and yellowness (b) values of the breast meat in broiler chickens during the summer months are associated with lower pH values and poorer water-holding capacity.^10^ The effective prevention of lipid peroxidation in poultry meat has been indicated by consumption of antioxidants. Special attention has been focused on plants, extracts and essential oils of herbs and spices to improve the quality of poultry products and foods.^11^ Turmeric is the rhizome of *Curcuma longa* *L*. and has beneficial effects on human health.^12^ Curcumin, demethoxycurcumin and bis-demethoxycurcumin are yellowish curcuminoids. These components are the main antioxidant compounds of turmeric.^13^ Curcumin has the ability to inhibit the lipid peroxidation and can scavenge the harmful free radicals.^14^ Furthermore, it increases the detoxification ability of glutathione- S-transferase enzyme.^14^ It has been reported that curcumin can enhance the blood alpha-tocopherol concentration in rat. This phenomenon increases vitamin E bioavailability.^15^^,^^16^ Moreover, curcumin has inhibited *in vitro* ROS production from activated macrophages.^16^ Cinnamon (C*innamomum zeylanicum*) is another beneficial medical herb. Cinnamaldehyde, cinnamyl acetate, cinnamyl alcohol, eugenol and carvacrol are the essential oils of cinnamon that have beneficial effects on health.^17^^,^^21^^,^^22^ Furthermore, cinnamon antimicrobial, antifungal and antioxidant properties have been reported previously.^17^^,^^18^ Cinnamaldehyde and eugenol possess antibacterial^19^ and antioxidant properties.^20^^,^^22^ Hence, this experiment was aimed to investigate the possible effects of C*innamomum zeylanicum *and* Curcuma lunga *powders in diet on nutrients composition, color and antioxidant properties of thigh meat in broiler chickens under heat stress condition.

## Materials and Methods

**Birds and husbandry.** Two hundred and fifty male 1-day-old broiler chicks (Ross 308) were housed randomly in 25 pens (1 m^2^). Continuous lighting program (23L: 1D) was used in the house. All the birds were fed the same starter (from day one to day 10 of age) and grower (from 11 to 24 days of age) mash form diets but received the different dietary treatments in the finisher period (from 25 to 42 days of age), (Table 1). All the diets (starter, grower and finisher) were produced via grinding (for the corn and soybean meal) by a hammer mill and mixing by a horizontal mixer. Feed and water were available *ad libitum*. The diets were formulated based on soybean-corn and treatments were control (recommended temperature according to Ross 308 catalog without supplement), heat stressed (32 ± 1 ˚C from 9:00 AM to 5:00 PM during finisher period day 25 to 42 of age and without supplement) and heat stressed birds fed 0.50% turmeric, 0.50% cinnamon and a blend of cinnamon and turmeric (0.25% turmeric + 0.25% cinnamon).^23^ Fresh Indian turmeric and cinnamon were purchased from a local market and included in the diets after grinding. The birds in the control group were raised in separate room under the recommended temperature of Ross 308. Average ambient relative humidity was 50.00% inside the house. 

**Sample preparation.** Fresh turmeric and cinnamon (containing 27.91 and 41.24 mg total phenolic compounds per g, respectively) were provided, ground and mixed with the diets. The Folin–Ciocalteu reagent method according colorimetry was used for total phenols determination. In this method, 1 g of dried plant tissue was extracted with 10 mL of 80.00% methanol. For this purpose, 0.50 mL of the extract, gallic acid (as the standard, Sigma-Aldrich, Steinheim, Germany), Folin (Sigma; diluted with water in the ratio 1:10) and 4.00 mL of sodium carbonate (Sigma; 1 M) solution were mixed and allowed to be kept at room temperature for 15 min. Standards with concentrations of 0, 25, 50, 100, 250 and 500 mg mL^-1^ were prepared and following incubation, the mixture was submitted to conventional spectrophotometry (model UV-160A; Shimadzu, Tokyo, Japan) in the range of 450 to 765 nm. Finally, total phenols were expressed as mg g^-1^ dry matter was obtained.^24^

**pH and nutrients composition test.** For the pH determination, approximately 2.50 g of ground thigh meat was homogenized in 25 mL of an iodoacetate solution inckuding 5 mM sodium iodoacetate (Sigma), 150 mM potassium chloride (Sigma) and the pH was adjusted to 7.00 with potassium hydroxide (Sigma) for 30 sec and the pH of the homogenate was determined using a pH meter (model TitroLine Easy; Schott Instruments, Mainz, Germany) that was calibrated at pH 4.00 and 7.00. Two pieces of meat from each left thigh were removed. One piece was used for proximate analysis of dry matter, crude protein, ether extract and ash contents according to the AOAC method.^25^ The other piece was used for determination of meat color indices. The samples were collected in plastic trays, weighed and stored in air tight plastic bags in a freezer (–20 ˚C) until laboratory analyses. Then, the meat samples were homogenized using a homogenizer (Model Ultra-Turrax T-25; Janke and Kunkel IKA-Labortechnik, Staufen, Germany) and analyzed.

**Table 1:** Composition of experimental diets

**Diet **	**Starter**	**Grower**	**Finisher**
***Ingredients (%)***			
**Maize**	32.99	34.45	39.28
**Wheat**	20.00	25.00	25.00
**Soybean meal (44% CP)**	39.33	33.50	28.23
**Soybean oil**	2.94	2.90	3.30
**Dicalcium phosphate**	2.10	2.15	2.15
**Lime stone**	1.10	0.86	0.86
**Lysine**	0.29	0.22	0.20
**DL-methionine**	0.38	0.08	0.14
**Vitamin-mineral mix** *	0.50	0.50	0.50
**Sodium chloride**	0.37	0.34	0.34
**Sand** †	-	-	0.50
**Total**	100	100	100
***Nutrients composition (%)***			
**Dry matter **	85.98	86.21	86.27
**Metabolizable energy (Kcal g** ^-1^ **)**	2860.00	2930.00	3000.00
**Crude protein **	21.99	19.99	17.99
**Fat **	3.87	3.93	5.37
**Fiber **	3.96	3.70	3.33
**Calcium **	1.00	0.90	0.89
**Available phosphorus **	0.45	0.45	0.44
**Chloride **	0.33	0.30	0.29
**Sodium **	0.16	0.15	0.15
**Methionine **	0.70	0.38	0.31
**Lysine **	1.32	1.23	1.09
**Arginine **	1.53	1.37	1.22
**Methionine + Cysteine **	1.07	0.73	0.73
**Tryptophan **	0.29	0.26	0.23
**Tyrosine **	0.98	0.89	0.81
**Threonine**	0.85	0.77	0.69

* Supplied per kilogram of diet: vitamin A, 9000 U; vitamin D3, 2000 U; vitamin E, 18 U; vitamin B12, 0.15 mg; riboflavin, 6.60 mg; calcium pantothenate, 10.00 mg; niacin, 30.00 mg; choline, 500 mg; biotin, 0.10 mg; thiamine, 1.80 mg; pyridoxine, 3.00 mg; folic acid, 1.00 mg; vitamin K3, 2.00 mg; antioxidant (Ethoxyquin), 100 mg; zinc, 50.00 mg; manganese oxide, 100 mg; copper, 10.00 mg; Fe, 50.00 mg; I, 1.00 mg; Se, 0.20 mg.

† The turmeric and cinnamon powders or the blend of both were replaced by sand in the diets at finisher

**2-thiobarbituric acid-reactive substances (TBARS) test.** For the TBARS determination, 10 g of minced thigh meat were weighed into a 50-mL test tube and 1 mL of 0.1% BHT was added. Then, 35 mL of 5% trichloroacetic acid (TCA) were added and the meat samples were homogenized at 13500 rpm for 30 sec. After filtering, 5 mL of the filtrate and 5 mL thiobarbituric acid (Merck, Darmstadt, Germany) solution (0.02 mM) were added to the test tube. Tubes were heated in a boiling water bath for 1 hr at 100 ˚C, cooled and then, absorbance was measured at 532 nm against a blank containing 5 mL of TCA and 5 mL of TBA solution. The TBARS values were expressed as mg per kg of sample.^26^

**Free radical scavenging activity (FRSA) test. **The FRSA was determined by 2,2’-azino-bis-3 ethyl benzothiazoline-6-sulfonic-acid (ABTS; Sigma) radical cation decolorization. The ABTS was dissolved in distilled water to a 7.00 mM concentration. The working solution was prepared by mixing two stock solutions of 7.00 mM ABTS and 2.45 mM potassium persulfate (Sigma) in equal amounts and allowed to react for 12 to 16 hr at room temperature in the dark to allow the completion of radical generation. This solution was then diluted with ethanol, so that its absorbance was adjusted to 0.70 ± 0.02 at 734 nm. The diluted ABTS solution (3.00 mL) was mixed with 30 mg meat sample and the absorbance was measured by a spectrophotometer (Novaspec II; Pharmacia LKB, Uppsala, Sweden) at 734 nm, using ethanol as a blank. The ABTS radical scavenging activity (%) was calculated by the following equation:^27^

$\mathrm{ABTS radical scavenging activity}\left(\%\right)=\frac{A_{\mathrm{blank}}-A_{\mathrm{sample}}}{A_{\mathrm{blank}}}\times 100$

**Meat color measurement. **The color of thigh meat was measured by a Chroma meter (model CR-400; Konica Minolta, Tokyo, Japan). Meat color ranged from light, L* > 53; to normal, 48 < L* < 51; and dark, L* < 46. Color categorization was based on 3 points of every meat in triplicate after slaughter.

**Statistical analysis. **The data were analyzed based on a completely randomized design using general linear model procedure of SAS (version 8.0; SAS Institute, Cary, USA). When treatment means were significant (*p *< 0.05), the Duncan multiple range test was used to compare the means. The experimental protocols were reviewed and approved by the Animal Care Committee at the Urmia University, Urmia, Iran. 

## Results

**Thigh meat nutrients composition. **The effect of dietary turmeric and cinnamon powders on thigh meat nutrients composition of broilers is shown in Table 2. Dietary supplementation of plants did not influence the thigh ash, protein and ether extract contents at day 42 of age (*p* > 0.05). Heat stress decreased the meat pH and DM values and consumption of all experimental diets (turmeric, cinnamon and both together) compensated these reductions in pH and DM due to heat stress to some extent but could not restore them to the same level as that of control treatment (*p* < 0.01). 

**Table 2:** Effect of cinnamon, turmeric and blend of both on thigh meat nutrient composition of broiler chicken on day 42 of age under heat stress condition

**Treatment**	**Ash (g kg** ^-1^ **)**	**EE (g kg** ^-1^ **)**	**CP (g kg** ^-1^ **)**	**DM (g kg** ^-1^ **)**	**pH**
**Control **	2.06	1.80	19.27	78.43^a^	6.88^a^
**HS**	2.08	2.05	18.63	74.36^c^	6.39^c^
**HS** ** + Turmeric **	2.11	1.77	19.41	76.33^b^	6.72^b^
**HS** ** + Cinnamon **	2.12	1.97	18.48	76.46^b^	6.64^b^
**HS** ** + Turmeric ** **‎** **+ Cinnamon **	2.10	1.84	18.93	76.25^b^	6.65^b^
**Pooled SEM**	0.03	0.06	0.14	0.36	0.04
***p*** **-value**	0.10	0.56	0.16	0.002	0.0001

HS: Heat stress, EE: Ether extract, CP: Crude protein, DM; Dry matter SEM: Standard error of the mean.

abc Data with different superscripts are significantly different within a dietary treatment (*p* < 0.01).

**Thigh meat color index and lipid peroxidation**. The effect of dietary turmeric and cinnamon powders on thigh meat color indices (lightness, redness and yellowness) is shown in Table 3. Heat stress did not affect meat redness and yellowness at day 42 of age but increased meat lightness and dietary supplementation of both plants numerically diminished the thigh lightness (*p* < 0.05). The effect of dietary turmeric and cinnamon powders on lipid peroxidation is shown in Table 4. Heat stress increased TBARS and RSA values (lipid peroxidation indices) but the blends of both plants returned these indices nearly to the control group (*p* < 0.05). 

**Table 3:** Effect of cinnamon, turmeric and blend of both on thigh meat color indices of broiler chicken under heat stress condition on day 42 of age

**Treatment**	**Lightness **	**Redness **	**Yellowness **
**Control **	39.83^b^	5.22	7.58
**HS **	45.03^a^	3.94	5.07
**HS** ** + Turmeric **	42.05^ab^	4.45	6.50
**HS** ** + Cinnamon **	42.55^ab^	4.24	5.54
**HS** ** + Turmeric ** **‎** **+ Cinnamon**	41.94^ab^	4.36	6.45
**Pooled SEM**	0.57	0.19	0.32
***p*** **-value**	0.05	0.32	0.11

HS: Heat stress, SEM: Standard error of the mean.

ab Means with different superscripts within each column are significantly different (*p* < 0.05).

**Table 4:** Effect of cinnamon, turmeric and blend of both on (meat or plasma) lipid peroxidation values of broiler chicken on day 42 of age under heat stress condition

**Treatment**	**FRSA (%)**	**TBARS (mg kg** ^-1^ **)**
**Control **	71.56^c^	0.78^b^
**HS**	81.88^a^	0.88^a^
**HS** ** + Turmeric **	77.27^ab^	0.82^ab^
**HS** ** + Cinnamon **	77.56^ab^	0.81^ab^
**HS** ** + Turmeric ** **‎** **+ Cinnamon ** **‎**	74.70^b^	0.80^b^
**Pooled SEM**	1.00	0.01
***p*** **-value**	0.00	0.04

FRSA: Free radical scavenging activity, TBARS: 2-Thiobarbituric acid-reactive substances, HS: Heat stress, SEM: Standard error of the mean.

abc Means with different superscripts within each column are significantly different (*p* < 0.01).

## Discussion

The reduced lightness (L (of thigh meat was detected by dietary supplementation of both plants alone or in combination. It has been accepted that heat stress stimulates glycolysis process and consequently decreases thigh pH quickly and this phenomenon causes the pale meat.^28^ Hot weather conditions of summer have increased the lightness and decreased the redness and yellowness of breast muscle in broilers.^10^ The yellowness of meat color during heat stress is related to ROS and lipid peroxidation.^10^

The elevated ROS production under heat stress takes place by disruption of the electron transport assembles in the membrane. Whereas, higher ROS production or lower ROS-scavenging elements cause severe oxidative damages to lipids, proteins and DNA.^29^ The supplementation of cinnamon and turmeric powders in current research prevented the thigh paleness. Although no research has been done on the effects of turmeric or cinnamon on meat quality in broiler chickens under heat stress, nevertheless the reduced meat lightness of broiler chickens by cinnamon and turmeric could have two reasons. First, the antioxidants properties of these plants may have retarded or inhibited the oxidation of substances by initiation or propagation of oxidizing chain reactions.^29^^,^^30^ These plants are the sources of natural antioxidants^31^^,^^32^ that can retard lipid oxidation. The heat stress reduced the pH in our experiment that was followed by the pallor color of meat. Both the cinnamon and turmeric powders stopped the pH loss, kept the acidity and hence prevented the pallor color of meat that could be the second possible reason of reduced meat lightness. These findings were confirmed by previous report^33^ that ground chicken meat with high pH is darker, redder and yellower than low pH meat. It has been reported that meat color is the most important factor of meat quality assessments by consumers and represents the meat freshness either. Changes in meat color are due to oxidation of red oxymyoglobin to metmyoglobin, which gives meat an unattractive brown color.^33^ Some reports have demonstrated the retardation of meat color loss by extending the red color and delaying the metmyoglobin formation by natural antioxidants.^31^^,^^34^^,^^35^ Although we observed lowered lightness by the phytogenic supple-ments in the recent study but inconsistent results have been reported by different phytogenic plants. For example, it has been noted that color parameters of raw pork patties did not vary by adding grape seed and bearberry extracts.^36^^,^^37^ The same results were obtained for fresh chicken breast meat with rosemary and sage essential oil at a concentration of 500 mg kg^-1^ diet.^38^ The use of oral garlic in the diet of pigs increased the pH of meat and reduced paint lightness, redness and yellowness in pork.^40^ In a study to evaluate the quality of broiler meat with green tea extract (0.10 and 0.20 g per kg), both the redness and yellowness indices were increased by the extract supplementation. These discrepancies may be due to the different plants of above mentioned researches and those used in our experiment. In our study, turmeric and cinnamon powders elevated the water-holding capacity and pH. Generally, pH value is a direct reflection of muscle acid content and affects the shear force, drip loss and color in meat. Muscle pH variation is also related to glycogen content of the muscle. It has been accepted that higher catecholamine secretion in response to an acute stressor prior to slaughter, increases glycogen breakdown and the rate of post-slaughter pH decline that causes the pale, soft and exudative meat.^39^ It has been reported that heat stress reduces the meat pH and stated that meat with a high pH has a higher water-binding capacity.^39^ Oxidative free radicals can cause oxidation of cellular components (lipid, DNA and carbohydrates) and consequently damage the function of normal cells. Lipid oxidation is the main reason of meat quality deterioration and adverse changes of flavor, color, texture and nutritive values.^40^ The thigh TBARS was increased by heat stress treatment in current experiment. It has been reported that oxidation of lipid components in muscle tissues is the major cause of low meat quality after slaughtering.^41^ The TBARS or malon-dialdehyde (MDA) are the soluble degraded products of lipids that reflect the amount of meat lipid oxidation.^41^ Heat stress induces oxidative stress and TBARS production.^43^ The blood TBARS concentration can reflect the body antioxidant capacity as well. It has been indicated that feeding 0.50 mg kg^-1 ^turmeric powder to broiler can significantly decrease the plasma concentration of MDA at heat stress conditions.^23^^,^^45^ It has been reported that supplementation of turmeric (with 90 ppm curcuminoids) trends to decrease the tissue MDA.^46^ These finding showed that dietary turmeric powder reduces the oxidative reactions in the body of broiler chicks and the rate of MDA production. The consumption of 0.50 mg kg^-1 ^cinnamon powder had significantly lowered the plasma MDA concentration during heat stress conditions in broiler.^44^ It has been shown that dietary cinnamon oil treatment (200 mg kg^-1^) decreases TBARS value in meat of quails and translated it to the cinnamon antioxidant properties.^46^ Studies have shown that cinnamon essential oils have antioxidant property.^18^^,^^48^ Cinnamon contains antioxidant compounds, which may be useful against free radical induced damages in cell membranes. Using 0.10%, cinnamon oil in broiler chicken diets decreased the MDA in plasma and duodenal mucosa and increased the glutathione peroxidase activity.^48^

In conclusion, the results of this study showed that dietary supplementation of turmeric and cinnamon plants together can diminish the MDA, FRSA and lightness, increase the pH and DM and consequently improve the meat quality deterioration during heat stress.
